# Albiflorin alleviates DSS-induced ulcerative colitis in mice by reducing inflammation and oxidative stress

**DOI:** 10.22038/IJBMS.2022.66678.14624

**Published:** 2023-01

**Authors:** Xiaohui Wang, Lianlin Su, Jinhua Tan, Tianwen Ding, Yinzi Yue

**Affiliations:** 1 Department of General Surgery, Bayinguoleng Mongolian Autonomous Prefecture People’s Hospital, Korla, 841000, Xinjiang, China; 2 School of Pharmacy, Nanjing University of Chinese Medicine, Nanjing, 210023, Jiangsu, China; 3 Department of General Surgery, Suzhou TCM Hospital Affiliated to Nanjing University of Chinese Medicine, Suzhou, 215009, Jiangsu, China; # These authors contributed eqully to this work

**Keywords:** Albiflorin, Dextran sulfate sodium, Mitogen-activated protein – kinase, NF-κB, Ulcerative colitis

## Abstract

**Objective(s):**

To clarify therapeutic potential of albiflorin and its intrinsic mechanisms against dextran sulfate sodium (DSS)-induced Ulcerative colitis (UC) mice.

**Materials and Methods:**

Sixty male C57BL/6 mice were randomly divided into five groups: negative control, positive, albiflorin low-dose group, albiflorin high-dose group and treatment control (Salicylazosulfapyridine “SASP”, 100 mg/kg) group. Acute colitis was induced in all groups except NC by administration of 3% DSS for 7 days. Albiflorin and SASP were administered via the intragastric route twice a day for 7 days. The changes of colon tissue were assessed by disease activity index (DAI), HE staining, and ELISA. Adrenodoxin expressions of UC colon tissues were evaluated by immunohistochemistry. Western blotting was performed to investigate related protein of the NF-κB and mitogen-activated protein kinase (MAPK) signaling pathways.

**Results:**

It has been found that the albiflorin shares similar influences as the SASP in ameliorating the DSS-induced UC. The reduced DAI and alleviated colon tissue damage were observed in albiflorin intervened groups. Moreover, albiflorin significantly inhibited myeloperoxidase activities and attenuated immuno-inflammatory response and elevated Foxp3 mRNA in colon tissue. Furthermore, investigations revealed that albiflorin could inhibit adrenodoxin isoform and activate activated phosphorylated NF-κB p65 and IκBα, which consequently suppressed phosphorylated p38 MAPK, extracellular regulated protein kinase (ERK), and c-Jun N-terminal kinase (JNK).

**Conclusion:**

These findings showed that albiflorin could alleviate DSS-induced murine colitis by activating inhibiting NF-κB and MAPK signaling pathways. It might be a potential therapeutic reagent for UC treatment.

## Introduction

Ulcerative colitis (UC) is a kind of inflammatory bowel disease (IBD), which is a chronic non-specific inflammatory disease related to rectum, colonic mucosa, and submucosa (1). Though UC has become a global disease, no effective treatment is presented with limited clinical intervention and anti-inflammatory and immunomodulatory drugs (2). Furthermore, the existing medications cannot fully control the progress of UC with high recurrence rate, mainly including steroidal and nonsteroidal anti-inflammatory drugs such as 5-aminosalicylate (5-ASA) or mesalamine (3). Previous studies have shown that the nonadherence rate was 41% after 5-ASA treatment, and the risk of recurrent rate was higher (4). Additionally, the treatment of 5-ASA is not accordant, and it is prone to result in a whole string of side effects, such as hepatorenal toxicity, drug dependence and easy to relapse after hormone withdrawal (5, 6). Therefore, it is urgent for scientists and herbalists to develop effective treatment methods for UC. 

In the Chinese Pharmacopoeia, paeoniflorin has been authorized as the only detection indicator of quality control for *Paeonia lactiflora *Pallas (family Ranunculaceae). *P. lactiflora* has been widely used to treat autoimmune diseases, including rheumatoid arthritis, Sjögren’s syndrome in Asian countries like China and Korea (7-9). Paeoniflorin, benzoylpaeoniflorin, hydroxy-paeoniflorin, paeonin and albiflorin can be extracted from the dried root of *P. lactiflora*, and the mixture collection of physiologically active ingredients is called total glucosides of paeony (TGP) (10). The pharmacological activity of albiflorin, a monoterpene glycoside, was paid little attention (11), though albiflorin reportedly exerts many pharmacological activities, including antioxidant, antidementia, and anti-inflammatory effects (12-14); research on albiflorin in UC remains limited.

NF-κB comprises a family of inducible transcription factors that regulate genes related to inflammatory response (15). The transaction from inactivated to activated states of NF-κB boosts the expression of inflammatory proteins, which trigger inflammation and oxidative stress (16). NF-κB comprises a family of inducible transcription factors that serve as important regulators of the inflammatory response. In its inactivated state, NF-κB combines with the inhibitory cytoplasmic protein IκB. Once activated, the p50/p65 subunits of NF-κB are transferred into the nucleus to boost the expression of inflammatory proteins, resulting in inflammation and oxidative stress (17). Persistent activation of NF-κB signaling has been detected in the mucosa of IBD patients and in murine IBD models (18, 19). It has been reported that inhibiting NF-κB by p65 will benefit IBD mouse *in vivo* (20). Increased mitogen-activated protein kinase (MAPK) activation has also been revealed to increase IBD incidence due to inducing iNOS expression (21). Taken together, NF-κB and MAPK signaling pathways are considered attractive therapeutic strategies to manage intestinal inflammation. The aim of this study is to determine the effects of albiflorin on colitis and investigate the underlying molecular mechanism *in vivo*.

## Materials and Methods

***Chemicals and reagents***

**Figure 1:**
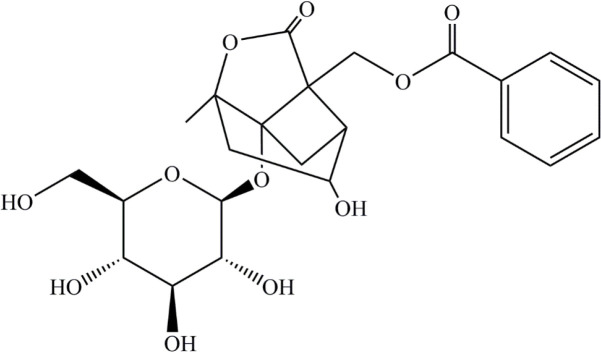
Chemical structure of albiflorin

Albiflorin (> 99% HPLC) was purchased from National Institutes for Food and Drug Control (Peking, R.P. China). The molecular formula is C_23_H_28_O_11_, and the chemical structure is shown as Figure 1. SASP (Sulphasalazine) was purchased from Sigma Aldrich (St. Louis, MO, USA). DSS was obtained from MP Biomedical (MW 36000-50000; Solon, OH, USA). Mouse TNF-α, IL-6, and IL-1β enzyme linked immunosorbent assay (ELISA) kits were purchased from R&D Systems (Minneapolis, MN, USA). The antibodies IκBα, phospho-IκBα (Ser32), phospho-NF-κB P65 (Ser536), Erk1/2, phosphoErk1/2 (Thr202/Tyr204), P38, and phospho-P38 (Thr180/Tyr182) were obtained from Cell Signaling Technology (Beverly, MA, USA). NF-κB, P65, JNK, phospho-JNK (Thr183/Tyr185), and adrenodoxin were provided by Signal way Antibody (College Park, MD, USA). GAPDH was purchased from Bioworld Technology (Louis Park, MN, USA). Griess Reagent was purchased from Beyotime (Shanghai, R.P. China). Trizol reagent was obtained from (Invitrogen, CA, USA). SYBR green dye was purchased from Vazyme Biotech (Jiangsu, China).

***Animals***

Male C57BL/6 mice 6–8 weeks of age were purchased from Shanghai Sipper-BK Lab Animal Co. Ltd. The animals were housed in plastic cages, four mice per cage, at a constant temperature (23 °C) and maintained in air-conditioned quarters with 12 hr light/dark cycles. The animals were acclimatized in the laboratory for 1 week before use. All animal welfare and experimental procedures were in accordance with and approved by the Research Ethics Committee of Suzhou TCM Hospital Affiliated to Nanjing University of Chinese Medicine. 

***Experimental procedures***

After acclimatization for 7 days, mice were randomly assigned into five groups based on the average body weight (n = 12 per group): negative control, positive group, albiflorin low-dose group (L-Alb, 50 mg/kg), albiflorin high-dose group (H-Alb, 100 mg/kg), and SASP group (100 mg/kg). Acute colitis was induced in all except the negative control group by administration of 3% (wt/vol, dissolved in drinking water) DSS (molecular weight 36-50 kDa) for 7 days. During the experimental period, albiflorin and SASP were given by oral gavage twice a day for 7 consecutive days. SASP was used as treatment control drugs. The animals were sacrificed on day 8, and the colons (from the caecum to the anus) were taken.

***Assessment of colitis***

Mice were weighed and measured daily for stool characteristics and bloody feces. The disease activity index (DAI) was measured by scoring body weight loss, feces status and bloody stools based on the scoring system (Table 1) according to Friedman *et al*. (22). 

***Evaluation of histological analysis***

On day 8 following induction of colitis, all mice were euthanized. The entire colon was removed from the cecum to the anus. For histopathological analysis, the rectal regions were fixed in 10% formalin, and paraffin-embedded sections were prepared for hematoxylin and eosin (H&E) staining. The pathological assessment was carried out according to Majumder *et al.* (23), with details shown in Table 2.

***Measurement of MPO, GSH, SOD, MDA activity, and cytokines concentrations***

Myeloperoxidase (MPO), glutathione (GSH), superoxide dismutase (SOD), and malondialdehyde (MDA) in colon were estimated spectrophotometrically using commercial kits. In addition, the concentrations of TNF-α, IL-1β, and IL-6 inflammatory factors in colon tissues were quantified by a direct ELISA technique using commercial ELISA kits (Thermo Fisher, USA) in accordance with the manufacturer’s instructions.

***RNA isolation and real-time PCR***

The total RNA was extracted from colon tissues using TRIzol reagent (Invitrogen, United States) according to the manufacturer’s recommendations, then it was quantified using NanoDrop 2000 (Thermo). Total RNA (1 mg) was reverse-transcribed to cDNA using PrimeScriptR RT reagent kit (TaKaRa Bio Inc). Real-time PCR was performed using Rotor-Gene Q (Qiagen) and SYBR_PremixR Ex TaqTM II (TaKaRa Bio, Inc.). The primers used in our study were shown in Table 3.

***Immunohistochemical (IHC) staining***

For immunohistochemical staining, the colons of mice were removed and immediately submerged in Methanol-Carnoy’s fixative at 4 °C for 3 hr. Then the tissues were treated with 100% methanol for 30 min, 100% ethanol for 20 min, and xylene for 15 min in turn. Fixed tissues were embedded in paraffin and cut into 4 mm sections. Subsequently, the sections were soaked in citrate buffer (pH 6.0) and heated by microwave for 20 min for antigen retrieval. The sections were blocked with a 5% BSA solution for 60 min and incubated with anti-adrenodoxin antibody (1:200; Abcam, Cambridge, UK) at 4 ℃ overnight. These sections were washed three times with PBS and incubated with biotinylated goat anti-rabbit or anti-mouse secondary antibody (Shanghai Jikai Gene Technology Co., Ltd.) at 37 ℃ for 20 min. Sections were washed three times again and incubated with 100 ml of substrate at room temperature for 10 min. The number of adrenodoxin positive cells was counted under the microscope at 400X magnification. Image-pro plus software was used to quantify the staining density. Based on the principles of equidistance, we randomly chose six views of distal end of the colonoscope in each section and quantified the averaged IOD of each section.

***Western blot analyses***

Colon tissues (100 mg each) were homogenized in ice-cold lysis buffer (RIPA, 1 mM PMSF, PMSF/RIPA=1/100). Homogenates were centrifuged at 12,000 × *g* for 15 min at 4 °C. The supernatants were collected and centrifuged again, and the final supernatants were collected for detection of p65, p-p65, p-IκBα, IκBα, p-p38, p38, p-ERK, ERK, p-JNK, JNK, Adx, TLR4, MyD88, Foxp3, and STAT5. Target proteins were detected using corresponding HRP-conjugated anti-rabbit IgG and antimouse IgG, as secondary antibodies. The signals were visualized using an ECL Western blot detection kit (Fdbio Science, China). The results were captured and quantified using the Carestream Molecular Imaging system (Carestream Health, Inc., USA). Nuclear and cytoplasmic extracts for western blotting were obtained by using a nuclear/cytoplasmic isolation kit (Solarbio^®^ Biotechnology, Beijing, China). Protein levels were determined using the BCA Protein Assay Kit (Thermo Fisher). Samples (50 μg each) were separated by denaturing SDS-PAGE and collected on a PVDF membrane (0.45 μm, Merck Millipore, USA) by electrophoretic transfer (Mini-Protean^®^ 3 Cell, Bio-Rad, USA). The membrane was pre-blocked with 5% BSA and 0.1% Tween-20 in Tris-buffered saline (TBST) and incubated overnight with a primary antibody (in TBST with 5% BSA). Each membrane was washed three times for 30 min and incubated with the secondary horseradish peroxidase-linked antibodies (Affbiotech, USA). Quantitation of detected bands was performed with the ImageQuant ^TM^ TL analysis software (General Electric, USA).

***Statistical analysis***

All the experimental data were shown as the mean ± standard deviation (SD). Statistically significant values were compared using the one-way ANOVA test and unpaired two tailed Student’s *t*-test (Graphpad Prism 6.0). Statistical significance was indicated when *P*<0.05, *P*<0.01, and *P*<0.001. 

## Results

***Effects of Albiflorin on clinical signs in UC mice***

**Figure 2.**
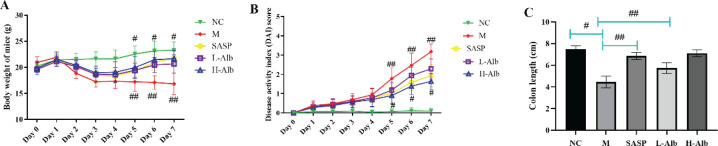
Albiflorin effectively attenuated the changes of body weight (A), scores of DAI (B), and colon length (C) in DSS-induced mice. Data are presented as means ± SD, n=12. Statistical independent samples one-way ANOVA test was used to assess the level of statistical differences between the groups: #=*P*<0.05 and ##=*P*<0.01 for comparison with the positive group

The UC mice model was induced by DSS, the symptoms were displayed as follows: (1) lower food consumption, increased water intake, and defecation frequency; (2) occult blood or even fecal blood appeared in feces on the 7th day; (3) 100% diarrhea during the whole experiment. The weight of mice gradually significantly decreased compared with the NC group. After treating with SASP or albiflorin, the rate of weight gain was higher compared with the positive control group, and the body weight of the NC group was stable (Figure 2A). DAI score of the positive control group was significantly higher than that of the NC group (*P*<0.01) (Figure 2B), while the DAI scores of SASP (20 mg/kg), L-Alb, and H-Alb groups were significantly lower than that of the DSS group (*P*<0.05). H-Alb and SASP treatment significantly rescued DSS-induced colon shortening compared with the positive control group (*P*<0.05) (Figure 2C).

***Histopathological findings***

**Figure 3:**
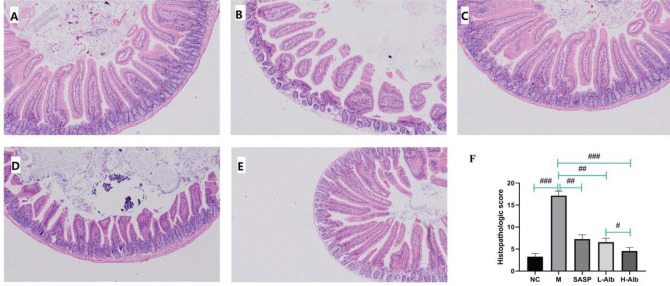
Representative colon H&E staining and the histological score at different groups (original magnification, 100 ×). A: negative control group, showing normal mucosa; B: positive group, showing mucosa atrophy and villi loss with the degeneration and necrosis of epithelium cells and infiltration of inflammatory cells; C: treatment control group, decreasing the DSS-induced infiltration of inflammatory cells and disruption of colonic architecture; D: albiflorin low-dose group, showing mucosa had better morphological structure; E: albiflorin high-dose group, showing mucosa had better morphological structure and infiltrated with less inflammatory cells. F: data of pathological score. Data are presented as means ± SD, n=12. #=*P*<0.05, compared with the albiflorin low-dose group, ##=*P*<0.01 and ###=*P*<0.001 compared with the positive group, were used to indicate significance

Mice in negative control group maintained normal colonic structures (Figure 3A). However, DSS treatment induced focal epithelial sloughing, a decrease in number of crypts, mucosal inflammation, and inflammatory cell infiltration (Figure 3B), so the histological scores were significantly higher than those of the negative control group (Figure 3F). Moreover, albiflorin also relieved the typical colon histological signs of colitis, including cellular infiltration of predominantly mononuclear macrophages and neutrophils, mucosal and submucosal lesion, and degeneration and necrosis of crypt cells (Figure 3D, E). Compared with mice of the positive group, L-Alb, H-Alb, and SASP significantly alleviated the pathological features of the colon and reduced the number of inflammatory cells. The villi of the colon in mice of the L-Alb/H-Alb and SASP groups were close to mice of the negative control group and smoother than those of the positive group.

***Effects of Albiflorin on oxidative stress and inflammatory responses in DSS-induced experimental colitis***

**Figure 4.**
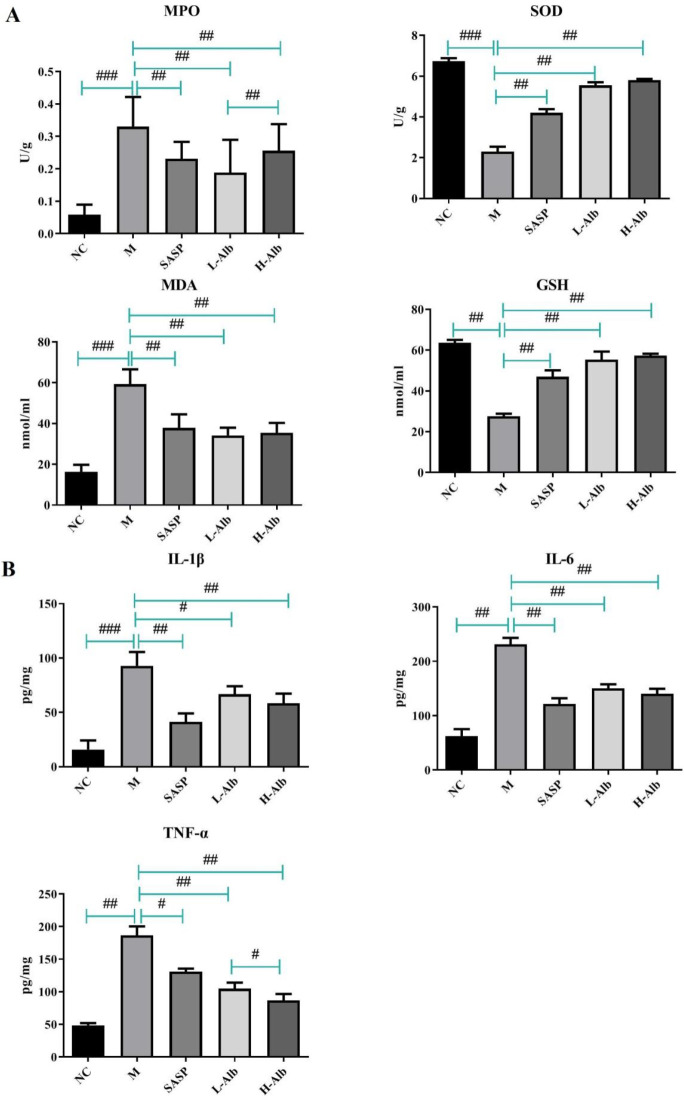
Effect of albiflorin on oxidative stress (A) and inflammation (B) in mice with DSS-induced colitis. Data are expressed as means±SD, n=12. #=*P*<0.05, compared with the albiflorin low-dose group, ##=*P*<0.01 and ###=*P*<0.001 compared with the positive group, were used to indicate significance

To investigate the correlation between the protection effects of albiflorin against the oxidative stress and inflammatory responses in DSS-induced colitis, the colon levels of MPO, GSH, SOD, MDA, TNF-α, IL-1β, and IL-6 were measured. Compared with mice of the negative control group, the levels of MPO, GSH, SOD, MDA, TNF-α, IL-1β, and IL-6 in mice of the positive group were significantly increased (*P*<0.01) (Figure 4). Interestingly, administration of the SASP and L-Alb, H-Alb could markedly reverse DSS-mediated changes. However, there was almost no significant differences between L-Alb and H-Alb (*P*>0.05).

***Effects of albiflorin on adrenodoxin, NF-κB, and TLR4 mRNA Foxp3 mRNA levels in colon of experimental mice***

**Figure 5:**
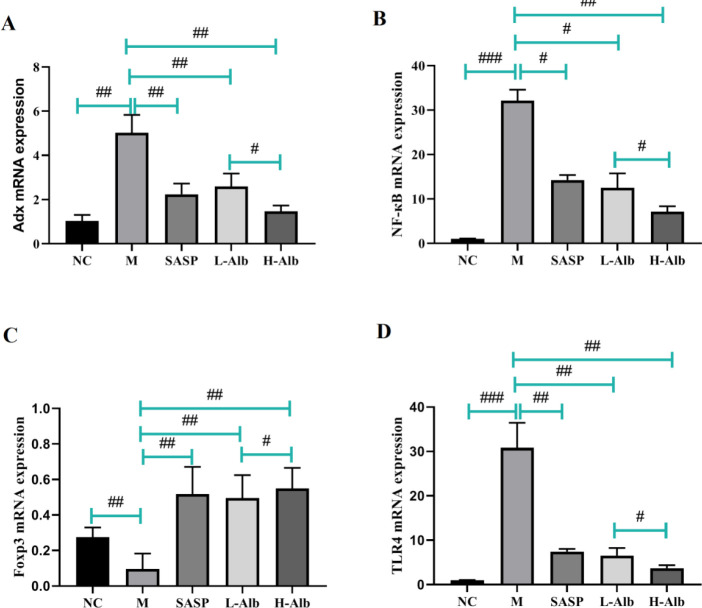
mRNA expression of adrenodoxin, NF-κB, Foxp3, and TLR4 was determined using real-time PCR in colonic tissues with DSS-induced colitis. Data are expressed as means±SD, n=12. #=*P*<0.05, compared with the albiflorin low-dose group, ##=*P*<0.01 and ###=*P*<0.001 compared with the positive group were used to indicate significance

RT-PCR was processed to further evaluate the inflammatory response, adrenodoxin, NF-κB, TLR4, and Foxp3 mRNA in the colon tissue were tested as shown in Figure 5. The results showed that the mRNA expression of adrenodoxin, NF-κB, and TLR4 in colonic tissues increased after DSS administration, while these up-regulations were suppressed by SASP and albiflorin treatment (Figures 5A, B, and D). Compared with the negative control group, Foxp3 mRNA in the DSS group was decreased in the DSS group (*P*<0.01). In contrast, Foxp3 mRNA in SASP and L-Alb, H-Alb groups were increased compared with DSS induced colitis mice (*P*<0.01 or *P*<0.05) (Figure 5C).

***Effects of albiflorin on adrenodoxin expression in colon tissues of ulcerative colitis mice***

**Figure 6:**
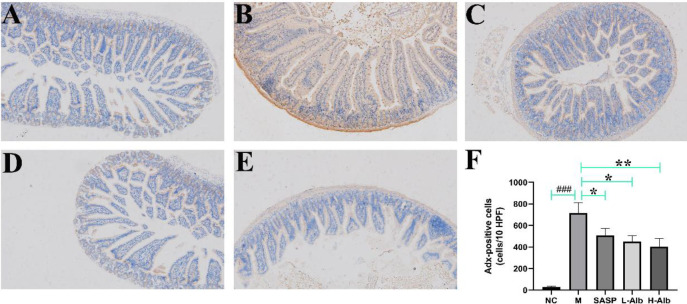
Immunohistochemistry (IHC) staining of colon tissue in DSS-induced UC mice showing the effects of albiflorin on oxidative stress. A-E: Adrenodoxin (brown-stained) is translocated from nucleus to the cytoplasm and extracellular site. F: Adrenodoxin-positive staining is represented as a composite score. Data are expressed as means±SD, n=12. *=*P*<0.05, compared with the treatment control or albiflorin low-dose group, **=*P*<0.01 and ###=*P*<0.001 compared with the positive group were used to indicate significance

In order to evaluate whether adrenodoxin was translocated from nucleus to cytoplasm and extracellular region in colitis, the effects of albiflorin on oxidative stress in DSS-induced UC mice were investigated by IHC expression of the adrenodoxin protein as an oxidative stress marker. IHC staining exhibited that adrenodoxin in the colon of UC mice (Figures 6A-E). As shown in Figure 6F, the albiflorin treatment remarkably decreased the levels of adrenodoxin protein expression by over 50% compared with the positive group. 

***Effects of albiflorin on regulation of adrenodoxin, Foxp3, TLR4, and STAT5 expression in colon tissues of ulcerative colitis mice***

**Figure 7:**
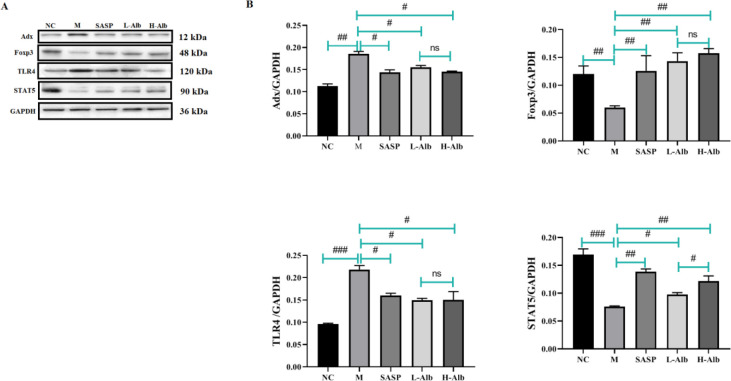
Effects of albiflorin on oxidative stress in DSS-induced ulcerative colitis. Oxidative stress-related molecules adrenodoxin, Foxp3, TLR4, and STAT5 were detected by Western blot. The level of total GAPDH was determined as loading control. All data are representative of 3 independent experiments. #=*P*<0.05, compared with the albiflorin low-dose group, ##=*P*<0.01 and ###=*P*<0.001 compared with the positive group were used to indicate significance GAPDH: Glyceraldehyde 3-phosphate dehydrogenase; DSS: Dextran sulfate sodium

The effect of albiflorin on the regulation of adrenodoxin, Foxp3, TLR4, and STAT5 in colon mucosa was analyzed using western blot as showed in Figure 7. Compared with the negative control group, adrenodoxin and TLR4 expression in DSS mice was increased (*P*<0.01). Compared with those in the positive group, adrenodoxin and TLR4 expression in SASP and L-Alb, H-Alb groups was reduced (*P*<0.01 or *P*<0.05). The levels of Foxp3 and STAT5 were significantly decreased in the positive group compared with the negative control group. Albiflorin-treated UC mice revealed a significant up-regulation of Foxp3 and STAT5. 

***Effects of albiflorin on DSS-induced activation of NF-κB and MAPK signaling in mice***

**Figure 8:**
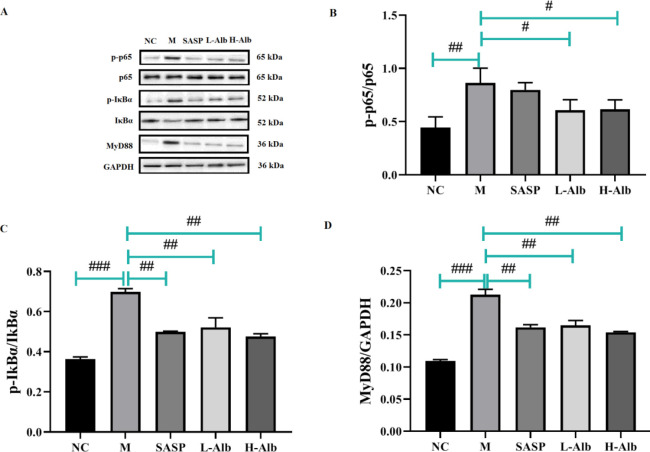
Albiflorin regulates the NF-κB pathways. (A) Expression levels of p-p65, p-65, p-IκBα, IκBα, and MyD88 were analyzed by Western blotting. (B-D) Analysis of protein concentration. The level of total GAPDH was determined as loading control. All data are representative of 3 independent experiments. Significant differences were indicated as #=*P*<0.05, compared with the albiflorin group, ##=*P*<0.01 and ###=*P*<0.001 compared with the positive group

GAPDH: Glyceraldehyde 3-phosphate dehydrogenase; NC: Negative control group; M: Positive group; SASP: Treatment control (Salicylazosulfapyridine) group

**Figure 9:**
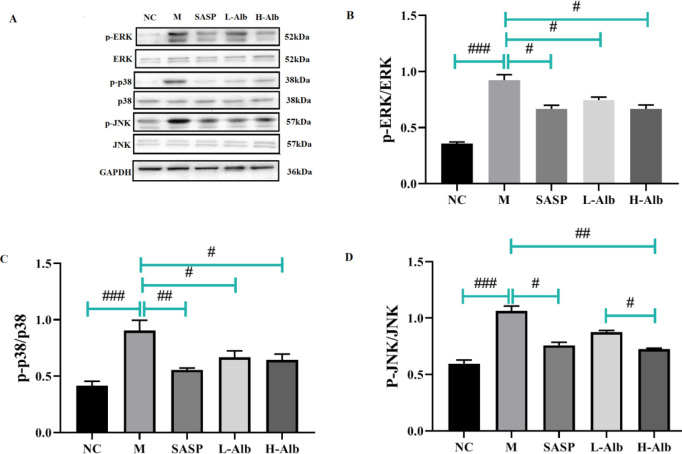
Effects of albiflorin on activation of MAPK signaling pathways in colonic tissues with DSS-induced colitis. (A) The expression levels of p-ERK, ERK, p-p38, p38, p-JNK, and JNK were analyzed by Western blotting. (B-D) Analysis of protein concentration. GAPDH was used as loading protein. All data are representative of 3 independent experiments. #=*P*<0.05, compared with the albiflorin low-dose group, ##=*P*<0.01 and ###= *P*<0.001 compared with the positive group were used to indicate significance

MAPK: Mitogen-activated protein kinase; ERK: Extracellular regulated protein kinase; GAPDH: Glyceraldehyde 3-phosphate dehydrogenase

NC: Negative control group; M: Positive group; SASP: Treatment control (Salicylazosulfapyridine) group

To further determine the anti-inflammatory mechanism of albiflorin, we detected the phosphorylation of p-p65, p-65, p-IκBα, IκBα, MyD88, p-ERK, ERK, p-p38, p38, p-JNK, and JNK through western blotting. The results showed that compared with that in the negative control group, the phosphorylation of p-65, p-IκBα, IκBα, MyD88, p-ERK, ERK, p-p38, p38, p-JNK, and JNK in the positive control group was significantly higher than in the NC group, which suggested that albiflorin significantly inhibited the phosphorylation of p-65 (Figure 8A, B), p-IκBα, IκBα (Figure 8A, C), MyD88 (Figure 8A, D), p-ERK, ERK (Figure 9A, B), p-p38, p38 (Figure 9A, C), p-JNK, and JNK (Figure 9A, D). The phosphorylation of ERK, p38, and JNK was significantly up-regulated in the colonic tissue of mice with DSS-induced colitis (Figure 9). 

**Table 1:** The standard scoring system of disease activity index (DAI) dextran sulfate sodium (DSS) induced ulcerative colitis (UC) mice

Score	Body weight loss	Feces status	Bloody stools
0	no loss	normal	no blood (no color within 2min)
1	0 -10%	loose stool (not attached to the anus)	presence (within 1- 2 min, fuchsia)
2	10% -15%	loose stool (attach to the anus)	presence (within 1 min, fuchsia)
3	15% - 20%	presence (within 1 min, fuchsia)	presence (within 1 min, fuchsia)
4	>20%	severe diarrhea	gross blood (instantly purplish blue)

**Table 2:** Histopathology grading system for colonic sections dextran sulfate sodium (DSS) induced ulcerative colitis (UC) mice

Feature graded	Grade	Description
inflammation	0	normal
	1	minimal infiltration of lamina propria, focal to multifocal
	2	mild infiltration of lamina propria, multifocal, mild gland separation
	3	moderate to mixed infiltration, multifocal with minimal edema
	4	marked mixed infiltration into submucosa and lamina propria with extensive areas of gland separation, enlarged Peyer’s patches, edema
epithelium	0	normal
	1	minimal: focal mucosal hyperplasia
	2	mild: multifocal tufting of rafts of epithelial cells with increased numbers of goblet cells
	3	moderate: multifocal to locally extensive epithelial attenuation or erosion with goblet cell hyperplasia
	4	marked: locally extensive to subtotal erosion or ulceration
glands	0	normal
	1	minimal: rare gland dilatation
	2	mild: multifocal gland dilatation
	3	moderate: multifocal gland dilatation with abscessation and occasional loss of glands
depth of lesion	0	none
	1	mucosa
	2	mucosa and submucosa
	3	transmural
extent of section affected	0	none
	1	minimal: <10%
	2	mild: 10–25%
	3	moderate: 26–50%
	4	marked: >50%

**Table 3:** Specific primers used in real-time PCR analysis dextran sulfate sodium (DSS) induced ulcerative colitis (UC) mice

Genes	Primer	Sequence (5′→3′)
Adx	FW	AGGCAATAGGTTTTGAGGGCCAT
	RV	TCCTCCCTGCTCCGATTCCG
Foxp3	FW	GAGAAAGCGGATACCA AATGA
	RV	GAGACAGAGATGGGCAAGAAG
TLR4	FW	GGTCAGACGGTGATAGCGAG
	RV	GGTCCAGGTTCTTGGTTGAG
NF-кB	FW	CCCCA CGAGCTTGTAGGAAAG
	RV	TAGTCCCC ACGCTGCTCTTCT
GAPDH	FW	CTGCAATCCGAAAGAAGCTG
	RV	ATCTTCAAACCTCCATGATG

UC: Ulcerative colitis; DSS: Dextran sulfate sodium

## Discussion

UC is a chronic inflammatory disease with an increasing occurrence and prevalence rates in developed countries (24). Even though patients who receive munosuppressants like azathioprine or methotrexate obtain certain therapeutic effect, side effects resulting from steroid dependence and serious infections impair their life equality and patients usually suffer from recurrence (25). It is reported that abnormal activation of NF-κB signaling pathway is closely associated with the UC progression (26). Various studies demonstrated that albiflorin has anti-inflammatory bioactivities, in current study, we evaluated the effects of albiflorin on anti-UC and elucidated the underlying mechanism.

The DSS-induced colitis model of mice has been widely used ***in vivo*** as human UC for its sharing same pathology with human IBD and availability of a quantitative scoring system. DAI, bodyweight loss, colonic length and histological scores are the main parameters used to determine the degree of UC (27). In this study, albiforin evidently improved the clinical symptoms of colitis mice by preventing the loss of bodyweights, extending the lengths of colon, down-regulating the infiltration of inflammatory cells and inhibiting the accumulation of MDA and MPO and secretion of inflammatory cytokines including IL-1β, IL-6 and TNF-α. Moreover, in this experiment, albiflorin dramatically increased the level of GSH and SOD, which suggested that the therapeutic effect of albiflorin on UC could be due to its antioxidant effect. In order to evaluate whether adrenodoxin was translocated from nucleus to cytoplasm and extracellular region in colitis, we next investigated the effects of albiflorin on oxidative stress in DSS-induced UC group. Existed study have shown that adrenodoxins refer to a subclass of ferredoxins that shuttle electrons from an NADPH-dependent adrenodoxin reductase to cytochrome P450, which is a part of steroid hormone biosynthesis and vitamin D metabolism (28). The data indicated that the albiflorin had a potential inhibition effect on oxidative stress in DSS-induced colitis, which was consistent with the attenuation of oxidative stress responses and inflammation as observed.

Current studies showed that Treg cell deficiency and Th17 cell increase contribute to the inflammation of UC (29). Signal transducer and activator of transcription (STAT) 5 and forkhead box P3 (Foxp3) are the key transcript factors for Treg cells (30), STAT3 are the key transcript factors for Th17 cells (31). The deficiency of Foxp3, a transcription factor depended by Treg cells’ development, induces severe inflammation in intestinal mucosa (32). We found that the expression of Foxp3 and STAT5 in colon mucosa were decreased, while adrenodoxin and TLR4 were increased, which implicates the dysregulation of Treg cell differentiation in colon mucosa. Albiflorin enhanced the expression levels of Foxp3 and STAT5 in colon, decreased adrenodoxin, NF-κB, and TLR4 mRNA with the increase of Foxp3 mRNA in colitis. The above phenomena revealed potential mechanism of Treg cells in colitis and the regulatory effect of albiflorin.

NF-κB is a ubiquitous transcription factor that regulates inflammatory cytokines. IκBα is an inactive form of NF-κB in the cytoplasm when unstimulated. Once activated, NF-κB translocates to the nucleus via phosphorylating and rapidly degrading IκBα (33). Activated NF-κB promotes the expression of various inflammatory genes (iNOS, COX-2, and TNF-α), including inflammatory cytokines involved in UC (34). Clinical studies show that activity of MAPKs was significantly increased during IBD intestinal epithelial injury. IHC revealed high expression levels of p38 MAPK in macrophages and neutrophils of the intestinal lamina propria (35). The expression of p-p38 MAPK was significantly higher in UC patients than in normal people, besides it presented a positive correlation with UC degree (36). Accumulating evidence suggests that the MAPK signaling pathway can affect the balance of inflammatory cytokines to preserve the health of the gastrointestinal tract, thus influencing the inflammatory process. 

For the purpose of studying the effect of albiflorin on NF-κB and MAPK signaling pathways in colonic tissue homogenates, we performed western blot analysis experiments. Our results demonstrated that albiflorin could inhibit adrenodoxin isoform and activate activated phosphorylated NF-κB p65 and IκBα, which consequently suppress phosphorylated p38 MAPK, ERK, and c-Jun N-terminal kinase (JNK). Similar outcome was observed where the inhibitory effects of albiflorin on MTX-induced enteritis were associated with the blockade of NF-κB activation (37). Together with our findings showing that albiflorin could strongly inhibit NF-κB activation and p-IκBα, p-p65, and pMAPKs production, these results indicate that albiflorin may be a prominent drug candidate for UC treatment.

This study expanded the spectrum of albiflorin’s anti-inflammatory activity, however, certain limitations need further explorations. First, albiflorin has been widely reported to have significant immune-regulating function, which may also be the mechanism of its anti-UC effect. Second, the improvement of albiflorin intervention on DSS-induced intestinal microbiota dysbiosis was uncertain. Third, *in vitro* study is needed to processed (e.g., albiforin affecting LPS-activated RAW264.7 cells) in further mechanistic studies.

## Conclusion

The present work demonstrated that albiflorin could ameliorate inflammation and oxidative stress of UC via activating adrenodoxin *in vivo*. Both outcomes of macroscopic and histological indicators displayed an overall improvement. Our results also exhibited a regulatory effect for albiflorin on NF-κB and MAPK signaling pathways *in vivo*. Albiflorin may be a potential pharmaceutical candidate for developing UC treatment.

## Authors’ Contributions

XW and YY Designed the experiments; XW and LS Performed experiments and collected data; JT and TD Discussed the results and strategy; XW, LS, JT, TD, and YY Approved the final version to be published.

## Conflicts of Interest

The authors declare no conflicts of interest.
